# Honokiol protects against doxorubicin cardiotoxicity via improving mitochondrial function in mouse hearts

**DOI:** 10.1038/s41598-017-12095-y

**Published:** 2017-09-20

**Authors:** Lizhen Huang, Kailiang Zhang, Yingying Guo, Fengyuan Huang, Kevin Yang, Long Chen, Kai Huang, Fengxue Zhang, Qinqiang Long, Qinglin Yang

**Affiliations:** 1 School of Basic Medicine, Research Center of Integrative Medicine, Guangzhou University of Chinese Medicine, 230 Guangzhou University City Outer Ring Road, Guangzhou, 510006 China; 2 Division of Cardiology, Department of Internal Medicine, Tongji Hospital, Tongji Medical College, Huazhong University of Science and Technology, 1095 Jiefang Ave, Wuhan, 430030 China; 3 Department of Nutrition Sciences, University of Alabama at Birmingham, 1675 Univ Blvd, Birmingham, AL 35205 USA; 4 Department of Cardiovascular Diseases, Union Hospital, Tongji Medical College, Huazhong University of Science and Technology, 1277 Jiefang Ave, Wuhan, 430022 China

## Abstract

Honokiol is a key component of a medicinal herb, Magnolia bark. Honokiol possesses potential pharmacological benefits for many disease conditions, especially cancer. Recent studies demonstrate that Honokiol exerts beneficial effects on cardiac hypertrophy and doxorubicin (Dox)-cardiotoxicity via deacetylation of mitochondrial proteins. However, the effects and mechanisms of Honokiol on cardiac mitochondrial respiration remain unclear. In the present study, we investigate the effect of Honokiol on cardiac mitochondrial respiration in mice subjected to Dox treatment. Oxygen consumption in freshly isolated mitochondria from mice treated with Honokiol showed enhanced mitochondrial respiration. The Dox-induced impairment of mitochondrial respiration was less pronounced in honokiol-treated than control mice. Furthermore, Luciferase reporter assay reveals that Honokiol modestly increased PPARγ transcriptional activities in cultured embryonic rat cardiomyocytes (H9c2). Honokiol upregulated the expression of PPARγ in the mouse heart. Honokiol repressed cardiac inflammatory responses and oxidative stress in mice subjected to Dox treatment. As a result, Honokiol alleviated Dox-cardiotoxicity with improved cardiac function and reduced cardiomyocyte apoptosis. We conclude that Honokiol protects the heart from Dox-cardiotoxicity via improving mitochondrial function by not only repressing mitochondrial protein acetylation but also enhancing PPARγ activity in the heart. This study further supports Honokiol as a promising therapy for cancer patients receiving Dox treatment.

## Introduction

Doxorubicin (Dox) is one of the anthracyclines that are effective anti-cancer drugs extensively used in clinical practice. One major side effect of this class of chemotherapeutic drugs is cardiotoxicity^1^, leading to dilated cardiomyopathy and heart failure^2^. A series of studies have proposed that reactive oxygen species (ROS) induced-mitochondrial damage was one of the major factors responsible for the cardiotoxic effect of Dox^3,4^. Dox-induced cardiac injury has been shown to correlate with mitochondrial dysfunction^5^, oxidative stress^6^, impaired DNA and protein synthesis, myofibril degeneration, and cardiomyocyte apoptosis^7^. Using antioxidants could partly protect cardiac cells from oxidative damage and cardiotoxicity^8^. Nevertheless, the clinical effectiveness of anti-oxidant therapies is still poor. Therefore, exploring novel therapeutic strategies to alleviate the cytotoxic effect of Dox remains a major challenge.

Honokiol is an active component extracted from the bark of Magnolia Officinalis, used widely in traditional Chinese medicine. In published literature, Honokiol has been shown to exert a wide spectrum of pharmacological effects, such as antitumor^9^, antibacterial^10^, antihypertensive^11^, and cardiac protection against pressure overload hypertrophy, Dox-cardiotoxicity^12,13^ and arrhythmia^14^. Earlier studies show Honokiol is an effective antioxidant that can scavenge free radicals and protect DNA^15^. Additionally, a previous study showed that Honokiol protects rat heart mitochondria against lipid peroxidation^16^. However, it remains unknown if Honokiol affects mitochondrial function in the hearts with *in vivo* treatment. The most recent finding that Honokiol protects the heart from Dox-cardiotoxicity^13^ emphasizes the importance of further defining the biological action of Honokiol in the heart to exploit its potential clinical applications.

In the present study, we focus on investigating how Honokiol treatment protects the mouse heart from Dox-induced mitochondrial dysfunction, oxidative stress, and inflammation via activating PPARγ.

## Results

### Honokiol protects mitochondrial respiration capacities in mice suffering Dox-induced cardiotoxicity

**Figure 1:**
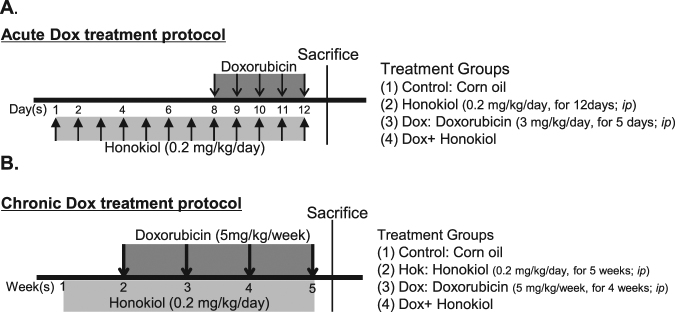
Experimental protocol for the acute (**A**) and chronic (**B**) treatments of Dox and pretreatment of Honokiol.

**Figure 2:**
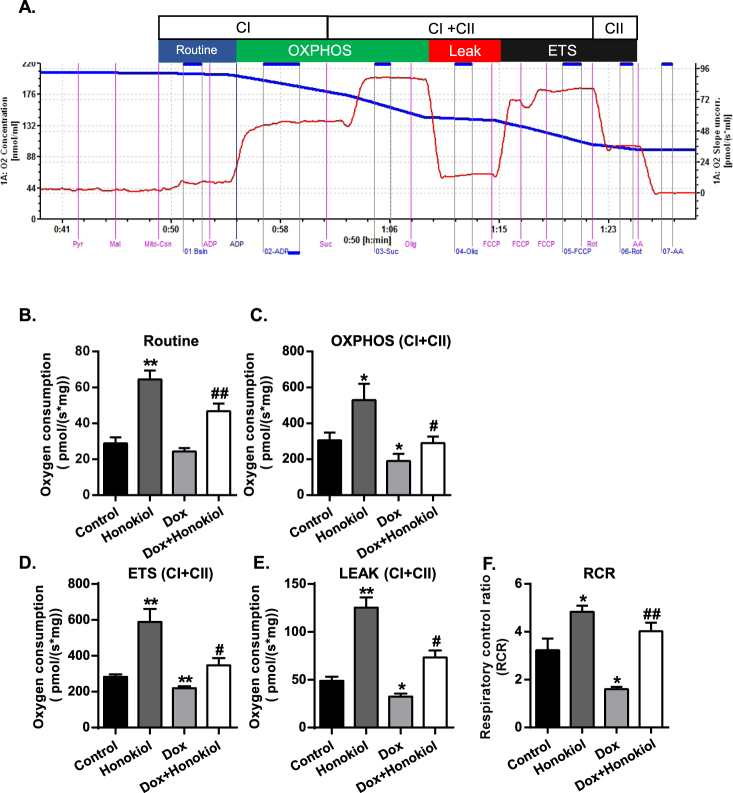
Honokiol protects mitochondrial respiration capacities in mice suffering Dox-induced cardiotoxicity. (**A**) Representative profiles of 6 different experiments in each group. (**B**) Basal mitochondrial respiration capacities (Routine), (**C**) Maximal phosphorylating respiration capacity via convergent input through complexes I and II (OXPHOS CI + CII). (**D**) The maximal uncoupled respiratory capacity of the ETS (ETS CI + CII). (**E**) Inhibition of the phosphorylation system by oligomycin (LEAK CI + CII). (**F**) Respiratory control ratio (RCR). n = 6, *p < 0.05, **p < 0.01 vs. Control group; vs. Dox group, ^#^p < 0.05, ^##^p < 0.01. Values are expressed as mean ± SEM.

To investigate the *in vivo* effects of Honokiol treatment on mitochondrial respiration, we freshly isolated mitochondria from mice of the four experimental groups as indicated (Fig. 1) and measured real-time oxygen consumption on these mitochondria in response to specific substrates and inhibitors using an Oroboro Oxygraph system (Fig. 2A). Routine mitochondrial respiration was established by the concomitant addition of malate (5 mM) and pyruvate (5 mM), followed by ADP (1 mM) and glutamate (5 mM), to measure the oxidative phosphorylation capacity of complex I (OXPHOS CI), driven by the NADH-related substrates^17^. The cardiac mitochondria of Dox showed no difference compared to control in Routine mitochondrial respiration (Fig. 2B). However, Honokiol-treatment markedly upregulated oxygen consumption compared to control and Dox-treated mice under the Routine condition (Fig. 2B). We then measured Maximal coupling respiration by adding a saturating concentration of ADP to assess maximal oxidative phosphorylation (OXPHOS CI + CII). The Maximal coupling respiration in cardiac mitochondria isolated from the Dox + Honokiol group was upregulated compared with vehicle control, and Honokiol treatment prevented the Dox-induced downregulation (Fig. 2C). The maximal uncoupled respiration of cardiac mitochondria was evaluated by adding FCCP (ETS CI + CII). Dox + Honokiol group showed similar upregulation of oxygen consumption in controlled mitochondria, and Honokiol treatment reduced the downregulation induced by Dox in cardiac mitochondria (Fig. 2D). LEAK CI + CII respiration measured by adding oligomycin was significantly increased in cardiac mitochondria from both groups of mice treated with Honokiol (Fig. 2E). We further analyzed the respiratory control ratios (RCR) to evaluate the structural integrity of the inner mitochondrial membrane (IMM) and OXPHOS efficiency. Consistently, Honokiol raised basal RCR, and attenuated Dox-induced RCR downregulation (Fig. 2F). These results demonstrate for the first time that Honokiol promotes cardiac mitochondrial respiration and improves impaired cardiac mitochondrial respiration by Dox in mice.

### Honokiol activates PPARγ signaling in cardiomyocytes

**Figure 3:**
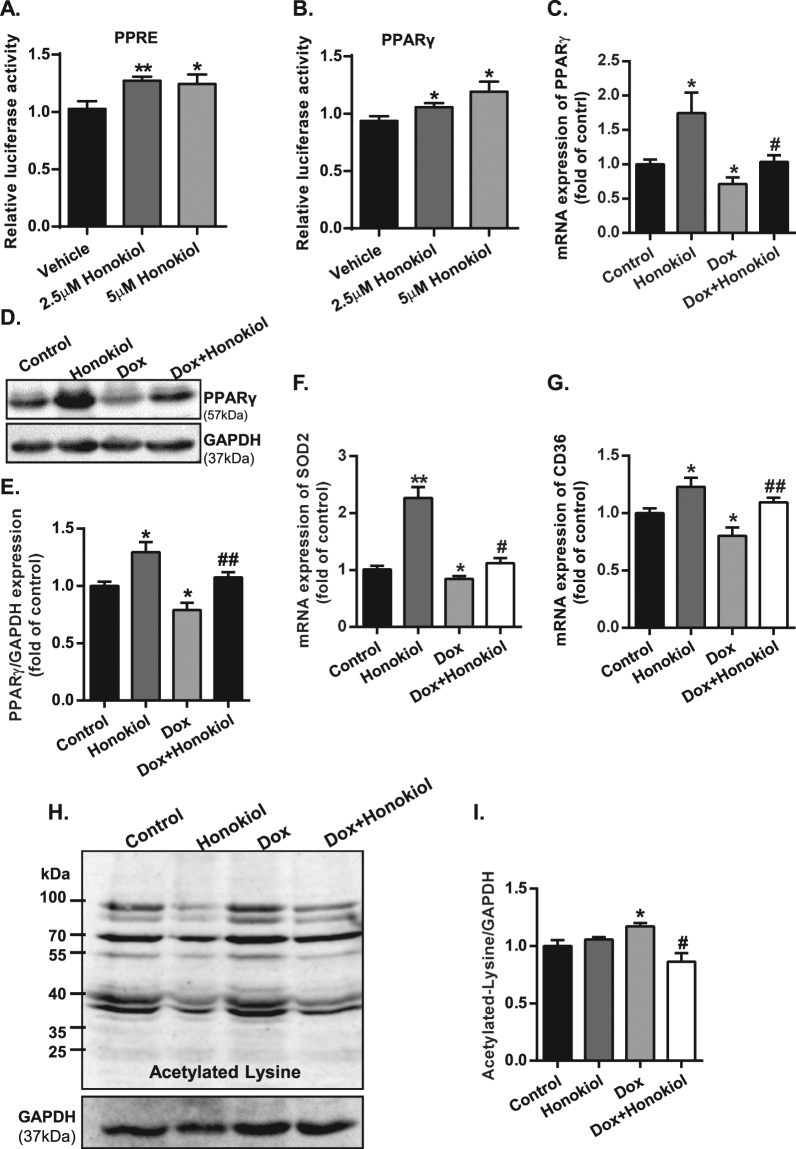
Honokiol activates cardiac PPARγ signaling. (**A** and **B**) H9c2 cells were transiently co-transfected with ACO-PPRE, PPARγ luciferase reporter gene and Renilla luciferase expression vector. 24 h after transfection, cells were treated with Honokiol (0, 2.5 μM, 5 μM) for one day. Then, cells were lysed and examined for reporter gene expression. The experiment shown above is representative of three independent experiments. (**C**) Relative expression levels of PPARγ mRNA were determined by Reverse transcription-PCR. (**D** and **E**) Protein levels of PPARγ relative to GAPDH in cardiac tissue homogenate. (n = 3–5). (**F** and **G**) Relative expression levels of SOD2 and CD36 mRNA were determined by Reverse transcription-PCR. (**H**) Cardiac tissue homogenate was prepared and analyzed for lysine-acetylation using anti-acetyl lysine antibody. (**I**) Quantification of relative lysine-acetylation (n = 3–5). *p < 0.05 vs. Control group; **p < 0.01 vs. Control group; ^#^p < 0.05 vs. Dox group; ^##^p < 0.01vs. Dox group. Values are expressed as mean ± SEM. Full-length images of blots and gels presented in supplementary information.

Honokiol has been reported as a natural PPARγ activator, a potential mechanism underlying the effect of Honokiol on cardiac mitochondria. To determine if Honokiol could activate PPARγ in cardiomyocytes, we first analyzed the effects of Honokiol on promoter activity via the PPAR response element (PPRE). In cultured embryonic rat cardiomyocytes (H9c2), luciferase reporter assay revealed that Honokiol increased the PPRE luciferase promoter activities at a dose of 2.5 μM (Fig. 3A). Furthermore, Honokiol treatment at both doses of 2.5 and 5 μM in the cultured H9c2 cells modestly enhanced the promoter activity of PPARγ (Fig. 3B). Moreover, *in vivo* treatment of Honokiol enhanced the transcript expression of PPAR γ in the heart (Fig. 3C). In mice with chronic treatment of Dox, cardiac PPARγ transcript was reduced by about 30% in Dox-treated hearts (Fig. 3C), which was rescued by Honokiol treatment (Fig. 3C). The expression of PPARγ protein in the heart showed the same pattern (Fig. 3D,E). We further examined the cardiac expression of PPARγ target genes, such as manganese super-oxide dismutase (SOD2) and Fatty acid translocase (CD36)^18–20^. Both SOD2 and CD36 were upregulated in the heart of Honokiol treated mice and Honokiol rescued the impaired SOD2 and CD36 expression in Dox-treated hearts (Fig. 3F,G). Supporting a recent report^12^, while Honokiol treatment had no effect on based protein acetylation, it did repress Dox-induced protein acetylation (Fig. 3H,I). These results indicate that Honokiol activates PPARγ pathway in the heart in addition to repressing stress-induced protein acetylation.

### Honokiol reduces myocardial reactive oxygen species levels in mice suffering chronic Dox-induced cardiotoxicity

**Figure 4:**
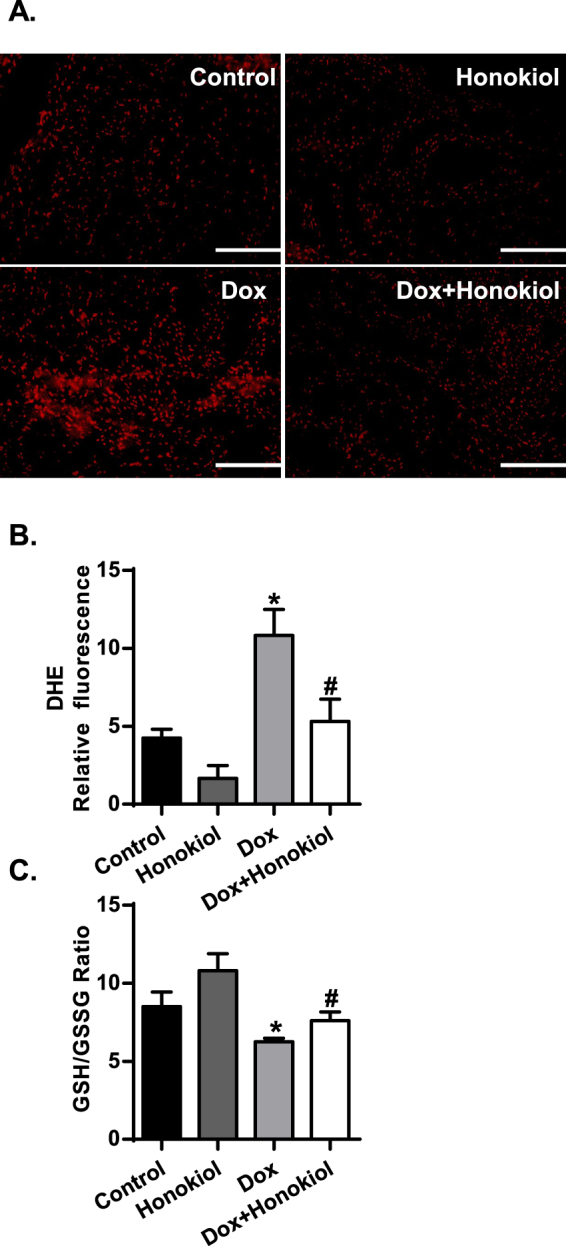
Honokiol reduced myocardial reactive oxygen species levels in mice suffering Dox-induced cardiotoxicity. (**A**) Representative images of Dihydroethidium staining (DHE, red). Scale bar, 100 µm. (**B**) Quantification of fluorescence density (n = 3). (**C**) GSH/GSSG ratio in cardiac tissue homogenates(n = 4). *p < 0.05 vs. Control group; ^#^p < 0.05 vs. Dox group. Values are presented as the mean ± SEM.

We next investigated the potential anti-oxidant effects of Honokiol as a PPARγ activator. Quantification of dihydroethidium (DHE) staining on heart sections showed that the intensity of DHE staining on heart sections of Dox-treated mice was markedly increased and the Dox-induced increase of DHE intensity was substantially repressed in heart sections from mice pretreated with Honokiol (Fig. 4A,B). Consistent with the DHE staining result, the Dox-induced decrease of GSH/GSSG rate, a result of rising oxidative stress^21^ was ameliorated by Honokiol pretreatment in mouse hearts (Fig. 4C). Therefore, these findings support that Honokiol exerts anti-oxidant effects to reduce Dox-mediated cardiotoxicity.

### Honokiol reduces chronic Dox-induced cardiac inflammation

**Figure 5:**
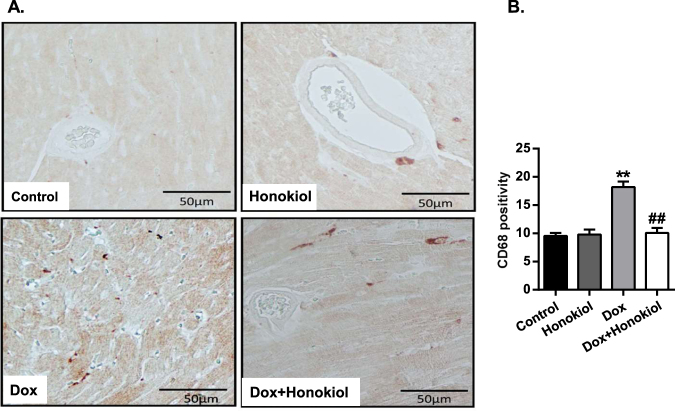
Honokiol reduces CD68-positive cells in Doxorubicin-mediated cardiotoxicity. (**A**) Representative images of CD68 immunohistochemistry on heart sections. Scale bar, 50 µm. (**B**) Quantitative analysis of CD68-positive cells. n = 4, **p < 0.01 vs. Control group; ^##^p < 0.01 vs. Dox group. Values are expressed as mean ± SEM.

We then investigated the effect of Honokiol, as a PPARγ activator and anti-oxidant, in alleviating Dox-induced cardiac inflammatory responses. Immunohistological staining of an inflammatory marker, CD68, on mouse heart sections revealed that Honokiol pretreatment largely abolished Dox-induced increase of CD68 positive stains on heart sections from Dox-treated mice (Fig. 5A,B). These results support that Honokiol exerts anti-inflammatory effects on mice with Dox-cardiotoxicity.

### Honokiol protects against side effects from both acute and chronic Dox treatments

**Figure 6:**
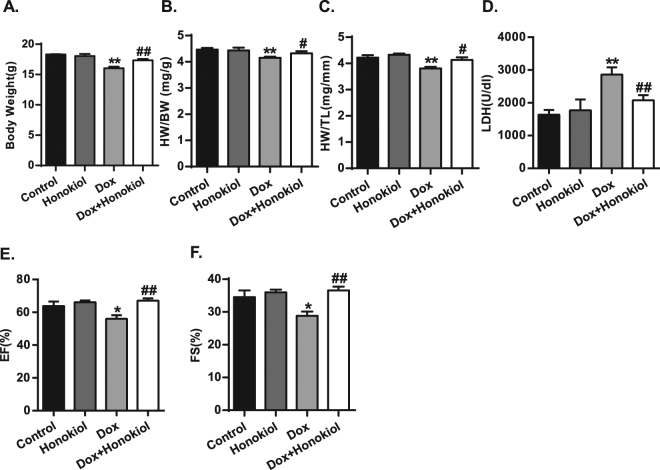
Honokiol improves cardiac dysfunction after acute Dox treatment. (**A**) Body weight. (**B**) Heart weight to body weight ratios. (**C**) Heart weight to tibial length ratios. (**D**) LDH content in blood samples. (**E**) Echocardiographic measurement of LV ejection fraction (EF%). (**F**) Echocardiographic measurement of fractional shortening (FS%).%). (n = 4–5). *p < 0.05 vs. Control group; ^# #^p < 0.01 vs. Dox group. Values are expressed as mean ± SEM.

We next validated the previous finding of protection by Honokiol against Dox-cardiotoxicity. In the Acute protocol study, Honokiol treatment rescued the body weight loss induced by Dox in mice (Fig. 6A). Acute Dox-induced cardiac atrophy was also rescued, evident by the restoration of heart weight to body weight ratio and heart weight to tibial length ratio (Fig. 6B,C). Echocardiography assessments confirmed that Honokiol treatment rescue the depression of cardiac contraction indicated by EF% and FS% (Fig. 6E,F). Furthermore, Honokiol treatment reduced the Dox-induced elevation of lactate dehydrogenase (LDH) activity (Fig. 6D) in mice subjected to acute Dox treatment. The severe illness in mice with Dox prevented echocardiographic measurement of cardiac function (Table 1).

**Table 1:** Honokiol improves cardiac dysfunction after acute Dox treatment.

	Control (n = 4)	Honokiol (n = 5)	Dox (n = 5)	Dox + Honokiol (n = 5)
LVPW;d (mm)	0.65 ± 0.03	0.63 ± 0.01	0.57 ± 0.02^*^	0.64 ± 0.02^#^
LVPW;s (mm)	1.06 ± 0.03	1.06 ± 0.04	0.94 ± 0.02^*^	1.1 ± 0.03^##^
LV Vol;d (µl)	80.31 ± 4.17	68.93 ± 3.71	56.47 ± 7.86^*^	60.94 ± 5.33^*^
LV Vol;s (µl)	30.44 ± 3.33	26.69 ± 2.43	24.41 ± 5.02	19.87 ± 1.88
LVID;d (mm)	4.24 ± 0.09	3.97 ± 0.09	3.62 ± 0.22^*^	3.76 ± 0.13^*^
LVID;s (mm)	2.82 ± 0.13	2.675 ± 0.11	2.351 ± 0.27	2.373 ± 0.09

Echocardiographic measurement in mice after acute Dox treatment. LVPW;d: Left Ventricular Posterior Wall, diastole; LVPW;s: Left Ventricular Posterior Wall, systole; LVID;d: Left Ventricular Internal Dimension diastole; LVID;s: Left Ventricular Internal Dimension, systole. n = 4–5. *p < 0.05 vs. Control group; **p < 0.01 vs. Control group; ^#^p < 0.05 vs. Dox group; ^##^p < 0.01vs. Dox group. Values are expressed as mean ± SEM.

**Figure 7:**
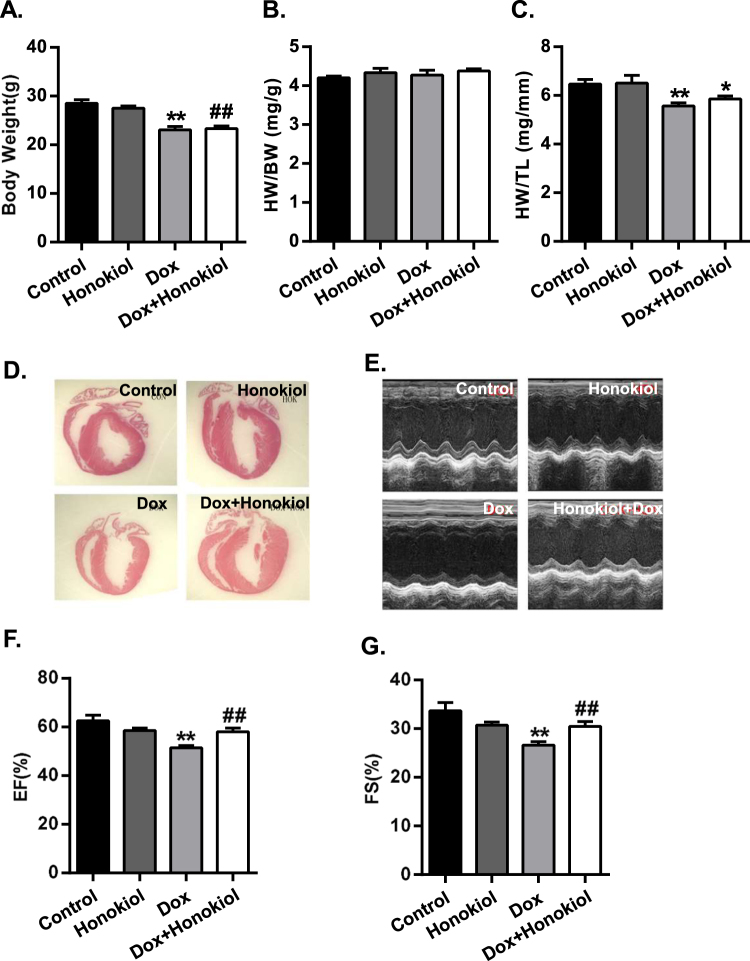
Honokiol improves cardiac dysfunction after chronic Dox treatment. (**A**) Body weight. (**B**) Heart weight to body weight ratios. (**C**) Heart weight to tibial length ratios (**D**) Representative longitudinal image of mouse hearts with HE staining. (**E**) Representative images of echocardiograms. (**F**) Echocardiographic measurement of LV ejection fraction (EF%). (**G**) Echocardiographic measurement of fractional shortening (FS%). (n = 7–13). *p < 0.05 vs. Control group; **p < 0.01 vs. Control group; ^#^p < 0.05 vs. Dox group; ^# #^p < 0.01 vs. Dox group. Values are expressed as mean ± SEM. Full-length images of blots and gels presented in supplementary information.

To gain clinically relevant insights, we focused on assessing mice with chronic Dox treatment. All mice from the chronic Dox treatment survived but with reduced body weight (Fig. 7A). The heart-to-body weight ratio (HW/BW) were similar among all the experimental mice (Fig. 7B). However, when comparing the heart weight to tibial length (HW/TL) ratio, mice with Honokiol treatment mitigated the Dox-induced HW/TL ratio decline (Fig. 7C). Histological and echocardiographic results support that Honokiol treatment reduced Dox-induced cardiac atrophy (Fig. 7D,E). Echocardiography showed that the Dox-induced decrease of ejection fraction (%EF) and fractional shortening (%FS) were significantly ameliorated in the Honokiol + Dox group (Fig. 7F,G).

**Figure 8:**
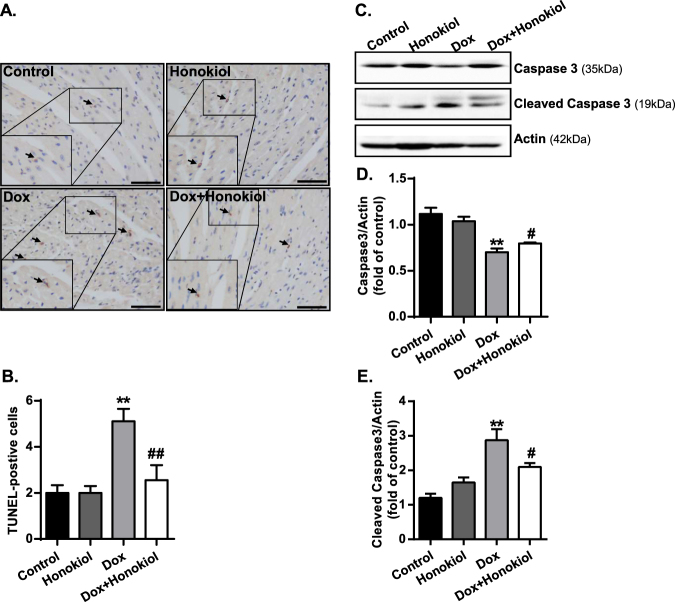
Honokiol protects cardiomyocytes against Dox-induced apoptosis *in vivo*. (**A**) Representative TUNEL images, High magnificent images of TUNEL positive cardiomyocytes were presented in the lower left. (Scale bar, 50 µm). (**B**) Quantitative analysis of myocardial apoptosis. The apoptotic nuclei were stained as brown. (**C**) Western blots of Caspase 3, cleaved caspase 3 and actin. (**D**) Protein levels of caspase 3 relative to actin in heart tissue homogenate. (**E**) Protein levels of cleaved caspase 3 relative to actin. (n = 3–5). **p < 0.01 vs. Control group; ^#^p < 0.05 vs. Dox group. Values are expressed as mean ± SEM.

TUNEL assays on heart sections revealed that honokiol significantly reduced Dox-induced cardiomyocyte apoptosis (Fig. 8A,B). Furthermore, Western blot analysis revealed that cleaved Caspase 3 in heart samples was increased in Dox-treated mice but was not as pronounced in mice with Honokiol treatment (Fig. 8C to E). Honokiol treatment prevented the Dox-induced reduction of left ventricular posterior wall thickness in diastole (LVPWd) and systole (LVPWs) (Table 2).

**Table 2:** Honokiol improves cardiac dysfunction after chronic Dox treatment.

	**Control** (n = 7)	**Honokiol** (n = 7)	**Dox** (n = 10)	**Dox + Honokiol** (n = 13)
LVPW;d (mm)	0.68 ± 0.04	0.65 ± 0.04	0.60 ± 0.06^**^	0.62 ± 0.04
LVPW;s (mm)	1.09 ± 0.07	1.03 ± 0.04	0.87 ± 0.06^**^	0.95 ± 0.08^#^
LV Vol;d (µl)	92.47 ± 17.26	84.68 ± 17.52	84.89 ± 12.85	83.52 ± 8.48
LV Vol;s (µl)	32.95 ± 10.033	34.86 ± 4.28	41.66 ± 8.12	36.56 ± 7.35
LVID;d (mm)	4.314 ± 0.37	4.31 ± 0.18	4.33 ± 0.27	4.31 ± 0.18
LVID;s (mm)	2.9 ± 0.39	2.99 ± 0.15	3.21 ± 0.24	3.04 ± 0.25

Echocardiographic measurement in mice after chronic Dox treatment. LVPW;d: Left Ventricular Posterior Wall, diastole; LVPW;s: Left Ventricular Posterior Wall, systole; LVID;d: Left Ventricular Internal Dimension diastole; LVID;s: Left Ventricular Internal Dimension, systole. n = 7–13. *p < 0.05 vs. Control group; **p < 0.01 vs. Control group; ^#^p < 0.05 vs. Dox group; ^##^p < 0.01vs. Dox group. Values are expressed as mean ± SEM.

Therefore, our results support that Honokiol protects the heart against Dox-induced cardiac dysfunction and pathological development.

## Discussion

The present study investigates the mechanisms of the cardio-protective effect of Honokiol against Dox-induced cardiotoxicity in mice. We provide evidence that Honokiol facilitates cardiac PPARγ expression and its activity, contributing at least partly to Honokiol’s role in improving mitochondrial respiration and reducing oxidative stress, inflammation and apoptosis in mouse hearts with Dox-cardiotoxicity.

Honokiol is a key component of a medicinal herb, Magnolia bark, which has been extensively used for thousands of years in traditional Chinese medicine. Previous research has shown that Honokiol is responsible for many pharmacological activities that may be beneficial for disease conditions such as cancer and cardiovascular disease. A recent study reported that Honokiol blocks and reverses cardiac hypertrophy in mice by activating mitochondrial SIRT3, subsequently increasing mitochondrial protein deacetylation^12,13^. Mitochondrial respiration in cultured cardiac fibroblasts was measured in the previous study^12^, but the cellular energetics in those fibroblasts did not optimally respond to oligomycin and FCCP. Moreover, majority of mitochondria in the heart are in cardiomyocytes. Therefore, our finding provide evidence supporting the *in vivo* role of Honokiol on cardiac mitochondrial respiration.

Dox is known to induce cardiac mitochondrial damage followed by oxidative damages. Lo *et. al* reported that Honokiol attenuated mitochondrial lipid peroxidation and reduced free radical scavenging activities^16^. On the other hand, Honokiol has been shown to induce mitochondrial dysfunction and swelling in isolated mitochondria^22^. However, this study was conducted by treating directly the isolated mitochondria extracted from rat liver with Honokiol, and the doses of Honokiol were relatively high. Recognizing the still obscured effect of Honokiol on mitochondrial function in the heart, we conducted a comprehensive analysis of the effects of Honokiol on cardiac mitochondrial energetics in mice with or without Dox treatment using the real time oxygraphy assessment. Another novel finding here is that Honokiol protects mitochondrial respiration capacities in mice suffering Dox-induced cardiotoxicity. By titration of various substrates, we stimulated the tricarboxylic acid (TCA) cycle and the different complexes of the electron transport chain. Routine and ADP-stimulated oxygen consumption rates, as well as maximal uncoupled oxidative capacity induced by FCCP (making oxygen consumption independent of ATP production), were ameliorated in cardiac mitochondria from Honokiol treated mice. These findings support that Honokiol enhances mitochondrial function in the *in vivo* animal, which in turn protects against Dox toxicity to mitochondria.

While Honokiol may protect mitochondria in Dox-treated hearts via de-acetylating mitochondrial proteins, other mechanisms may also be involved. A previous study showed that Honokiol binds to the PPARγ ligand-binding domain (LBD) and acts as a partial agonist in a PPARγ-mediated luciferase reporter assay^23^. The study further showed that Honokiol might work as a modest PPARγ activator without inducing adipogenesis^23^. A most recent report also shows that Honokiol attenuates diet-induced nonalcoholic steatohepatitis by regulating macrophage polarization through activating PPARγ^24^. Our study on cultured rat embryonic cardiomyocytes (H9c2 cell) confirms a mild, but significant, effect of Honokiol in activating the transcriptional activity of PPARγ. Recognizing the limitations of interpreting results from cultured H9c2 cells, which are not fully differentiated cardiomyocytes and with low PPARγ expression^25–28^, we further assessed the effect of Honokiol on cardiac expression of PPARγ with the treatment of Honokiol in mice. Consistently, both transcript and protein expression of cardiac PPARγ were upregulated in mice with Honokiol treatment. The upregulation of cardiac PPARγ by Honokiol is not attenuated by Dox treatment. The modest effects of Honokiol on the PPRE reporter assay in cultured H9C2 cell may be due to the relative low expression of PPARγ in H9C2. However, it appears that Honokiol treatment *in vivo* is sufficient to induce substantial upregulation of cardiac PPARγ transcript and protein. Although other signaling pathways, such as epigenetic modifications of Sirt3, may be involved, the PPARγ upregulation and activation effects of Honokiol appear to be the key factor in enhancing the cardiac PPARγ expression and activity. It is well established that PPARγ activation could increase gene expression of mitochondrial metabolic genes, thus facilitating mitochondrial respiration. Therefore, PPARγ activation may contribute to the mitochondrial effect of Honokiol treatment.

Given that mitochondria are the major organelles that produce ROS^29^, Dox-induced mitochondrial dysfunction causes the generation of excessive ROS in the cardiac tissue^30^. Yu *et al*. reported that Honokiol protects against renal ischemia/reperfusion injury by suppressing oxidative stress, iNOS, inflammation, and STAT3 in rats^31^. In the present study, Honokiol treatment depressed total ROS levels, which illustrated by the less pronounced decreased ratio of GSH/GSSG in mice suffering from Dox-induced cardiotoxicity. Furthermore, the anti-oxidative effects of Honokiol appear to attribute to its mitochondrial respiration enhancing and uncoupling capabilities^29^. As a result, cardiac inflammation-induced by Dox was attenuated by Honokiol pretreatment. The anti-oxidative and anti-inflammation capacities of Honokiol apparently contribute to its cardiac protective effect against Dox-cardiotoxicity. Moreover, the anti-oxidative stress effects of Honokiol may also derive from its transcriptional regulation of endogenous anti-oxidants as a PPARγ ligand in the heart as we reported previously^18^. On the other hand, it has been well documented that PPARγ-specific ligands exert anti-inflammatory effects in the cardiovascular system^32^. Activating PPARγ should contribute to Honokiol anti-inflammatory effects in mice treated with Dox.

In addition to the cardiac protective effects, the potential beneficial effects of Honokiol on multiple tissues in the body are possible, which are especially obvious in those mice subjected to the acute Dox treatment. On the other hand, previous studies suggest Honokiol may be a promising therapeutic anti-cancer agent. Lu *et al*. reported that Honokiol induces cell cycle arrest and apoptosis *in vitro* and *in vivo* in human thyroid cancer cells^33^. Hua *et al*. reported that Honokiol augments the anti-cancer effects of oxaliplatin in colon cancer cells^34^. Therefore, the potential dual effects of Honokiol on anti-cancer and cardiac protection indicate the promising clinical benefits of Honokiol treatment among cancer patients. Further preclinical and clinical studies are warranted.

In conclusion, our study demonstrates for the first time that Honokiol treatment protects the heart from Dox-cardiotoxicity via facilitating mitochondrial respiration and exerting anti-oxidant and anti-inflammation effects, not only by repressing mitochondrial protein de-acetylation but also activating PPARγ signaling. Honokiol may be a promising drug or supplement in cancer patients subjected to Dox chemotherapy.

## Methods

### Animal study

All animal studies were approved by the Animal Research Committee of Tongji Medical College, Huazhong Science and Technology University, China. All animals were bred at the animal care facility of Tongji Medical College under specific pathogen-free conditions. Mice were housed in temperature-controlled cages under a 12-h light–dark cycle and given free access to water and normal chow. All animal experiments were in accordance with the National Institute of Health Guide for the Care and Use of Laboratory Animals, and were approved by the Committee on the Ethics of Animal Experiments of Tongji Medical College. Male C57BL/6 J mice (Beijing HFK Bioscience CO. LTD) at their ages of 8 weeks were allocated into 4 groups: Control vs honokiol treated mice with and without doxorubicin (CAS No: 25316-40-9, Dalian Meilun Biotechnology Co. Ltd.) acute (3 mg/kg/day for 5 days, *i.p*.) and chronic treatments (5 mg/kg/week for 4 weeks, *i.p*.). Honokiol (CAS No: 35354-74-6, MedChem Expression, USA) treatment (0.2 mg/kg/day for 35 days, *i.p*.) started one week before the start of Dox treatment. The detailed protocol is indicated (Fig. 1). Dox was dissolved in 0.9% normal saline, and Honokiol was dissolved in corn oil. In control mice, vehicle (corn oil) was used. After echocardiographic assessment, all animals were sacrificed, and organs were collected and snap frozen in liquid nitrogen followed by storage at −80 °C. Hearts were also perfused with cardioplegia solution (25 mM KCl and 5% glucose) and fixed with formalin for histological analysis as described previously^35–37^.

### Echocardiographic assessment

Echocardiography was performed in mice anesthetized with 1.5% isoflurane as previously described^37^ using a Vevo 1100 Imaging System (Visual Sonics, Toronto, Canada) equipped with a 30 MHz linear-array transducer. The following parameters were obtained: LV end-systolic diameter (LVESD) and LV end-diastolic diameter (LVEDD), the percentage of fractional shortening (FS, %), ejection fraction (EF, %), and other parameters were measured from the M-mode images and two-dimensional obtained in the long- and short-axis views by the corresponding matching software. All measurements were performed from leading edge according to the American Society of Echocardiography guidelines.

### Mitochondrial isolation

Cardiac mitochondria were isolated from male C57BL/6 J mice. Briefly, blood and main vasculature were dissected, and heart tissue was minced on ice, then suspended in buffer A (250 mM sucrose, 10 mM Tris/Cl, 0.5 mM EDTA) and homogenized using a 2 ml Potter-Elvehjem Teflon-glass homogenizer. The resulting samples were centrifuged at 1000 g for 10 minutes, and the supernatant with mitochondria was poured into another ice-cold tube, followed by centrifugation at 8000 g for 5 minutes. The mitochondrial enriched sediments were resuspended in buffer A. Mitochondrial protein content was determined by the Lowry method.

### Assessment of mitochondrial respiration

Mitochondrial respiration was measured as previously described^17^. Briefly, 200 μg of freshly isolated mitochondria were measured in 2 ml of MirO5 mitochondrial respiration medium (3 mM MgCl_2_, 60 mM Lactobionic acid, 20 mM Taurine, 10 mM KH_2_PO_4_, 20 mM HEPES,110 mM D-sucrose, 1 g/L BSA and 0.5 mM EGTA) using an Oroboros 2k-Oxygraph (Oroboros Instruments, Innsbruck, Austria). Mitochondrial respiration was stimulated by basal substrates (5 mM pyruvate, 5 mM malate, 10 mM glutamate and 1 mM ADP) for complex I activity. Next 10 mM succinate was added to measure combined respiration rates of complex I and complex II. After that, 2 μg/ml oligomycin was added for the estimate of the overall mitochondrial related respiration. Further, carbonylcyanide p-trifluoromethoxyphenylhydrazone (FCCP) was added to determine the maximal coupling respiration. Finally, the addition of antimycin A allowed for the measurement of non-mitochondrial oxygen consumption.

### LDH activity

Serum was separated from the blood for the measurement of tissue injury marker lactate dehydrogenase (LDH)^38^ using an LDH assay kit (Applygen Technologies Inc, Beijing, China). The assay is based on LDH-dependent and NADH-catalyzed reduction of the tetrazolium salt 3-(4,5-dimethylthiazol-2-yl)-2,5-diphenyltetrazolium bromide to a reduced form. Absorbance was measured at 440 nm using a BioTek plate reader (BioTek), and the values were directly proportional to the enzyme activity.

### Detection of ROS production

Dihydroethidium (DHE, Beyotime Institute of Biotechnology, Haimen, Jiangsu, China) was applied to frozen section samples (7 μm-thick sections). The heart sections were stained with 5 μM DHE and incubated in a light-protected humidified chamber at 37 °C for 30 min. Fluorescence intensity was examined by fluorescence microscopy (Nikon DXM1200 fluorescence microscope) and images were analyzed with the ImageJ software.

### GSH/GSSG ratio assay

The ratios of glutathione and oxidized glutathione (GSSG) were measured using a GSH and GSSG assay kit (S0053, Beyotime Institute of Biotechnology), according to the manufacturer’s protocol.

### Cell culture

H9c2 rat cardiomyocytes (ATCC) were cultured in Dulbecco’s Modified Eagle’s Medium (DMEM) with 10% fetal bovine serum, 100 U/mL penicillin, and 100 μg/mL streptomycin. The cells were grown at 37 °C and 5% CO_2_.

### Luciferase Reporter Assay

H9c2 cells (0.5 × 10^5^ cells per well) were plated in 24-well culture dishes and maintained overnight at 37 °C with 5% CO_2_. H9c2 cells were transiently co-transfected with PPARγ, ACO-PPRE and Renilla luciferase expression vector using Lipofectamine™ 2000 Reagent (Invitrogen, USA). After 6 h incubation with transfection mixtures, the culture medium was replaced by 10% FBS medium. Twenty-four hours after transfection, cells were treated with Honokiol (0, 2.5 μM, 5 μM) for one day. Finally, cells were harvested in a centrifuge tube and centrifuged at 1000 g for 5 minutes. The luciferase activity was measured in a luminometer using dual-luciferase reporter assay system (Promega) according to the manufacturer’s instructions. Transfection efficiency was normalized by Renilla luciferase reporter (pRL-CMV vector, Promega).

### Apoptosis Detection by TUNEL Assay

Apoptosis was assessed in heart sections using the terminal deoxynucleotidyl transferase-mediated nick-end labeling (TUNEL) of fragmented nuclei assay. According to the manufacturer’s instructions (*in situ* Cell Death Detection Kit, POD; Roche, Mannheim, Germany), the paraffin-embedded sections of heart tissues of different groups were processed. Apoptotic cell number in each section was calculated by counting the number of TUNEL-positive apoptotic cells in 5 fields per slide randomly at 400x magnification.

### Total RNA Extraction and RT-PCR

Total RNAs were extracted using RNA simple Total RNA Kit (TIANGEN, Beijing, China). Total RNA (1 µg) were reverse-transcribed into cDNA using a cDNA Synthesis Kit (TRANSGEN, Beijing, China) according to the manufacturer’s protocol. PCR products were separated on 2% agarose gels and documented with BioRad Gel Doc. Results from each gene/primer pair were normalized to β-actin and compared across conditions. The sequences of the primers are listed as follows:

PPARγ forward primer 5′-AAAGACCCAGCTCTACAACA-3′ and reverse primer 5′-TCGTAGATGACAAATGGTGA-3′, SOD2 forward primer 5′-GCCTCCCAGACCTGCCTTAC-3′ and reverse primer 5′- TCGGTGGCGTTGAGATTGT-3′, CD36 forward primer 5′-AGATGACGTGGCAAAGAACAG-3′ and reverse primer 5′-CCTTGGCTAGATAACGAACTCTG-3′ β-actin forward primer 5′- CTGTCCCTGTATGCCTCTG-3′ and reverse primer 5′-ATGTCACGCACGATTTCC-3′. At least three independent experiments were conducted to ensure the reproducibility of the data.

### Western blotting

The frozen cardiac tissues were lysed in a RIPA buffer (Applygen Technologies Inc, Beijing, China). The BCA Protein Assay Kit (Boster Biological Engineering Co., Ltd, Wuhan, China) was used to measure protein concentrations. Thirty microgram protein samples were separated by 10% sodium dodecyl sulphate-polyacrylamide (SDS-PAGE) gel electrophoresis and transferred to a PVDF membrane (EMD Millipore, Billerica, MA, USA). Furthermore, 5% non-fat dried milk was used to block the membrane for 2 h at room temperature and then incubated with primary antibodies (PPARγ 1:1,000, Santa Cruz, USA; Acetylated-Lysine 1: 1,000, Cell Signaling) at 4 °C overnight. After being washed with TBST, the membrane was incubated with horseradish peroxidase-conjugated secondary antibody anti-mouse IgG (1: 2,500, Boster Biological Engineering Co., Wuhan, China) and peroxidase-conjugated secondary antibody anti-rabbit IgG (1:10,000, Santa Cruz, USA) for 1 h at room temperature. Enhanced chemiluminescence reagents were used to exposed the bands. Equivalent protein loads were verified and normalized using GAPDH (1:1,000, Santa Cruz, USA) or actin (1:1,000, Sigma-Aldrich, USA) blots. Finally, bands were then quantified by densitometry using ImageJ software.

### Histological analysis

Heart tissue samples were fixed in 4% formalin for immunohistochemistry. Briefly, samples were embedded in paraffin, cut into 4 μm-thick sections, and stained with rabbit anti-CD68 (1:100, Boster, Wuhan, China). Staining was visualized with biotin labeled Goat anti-rabbit Ig-G secondary antibody. Staining with the secondary antibody alone was performed as a negative control. The number of CD68-positive cells was similarly counted in 5 randomly selected fields at 400x magnification and used to calculate the mean number of positively stained cells per microscopic field. Images were taken with a light microscope.

### Statistical Analyses

Data for 2-group comparisons were analyzed with the nonparametric Student *t*-test; otherwise, data were analyzed by one-factor or mixed, 2-factor ANOVA and multiple comparisons test using the GraphPad Prism 6 software (GraphPad Software Inc.). Values of Quantitative results were expressed as mean ± SEM. Differences between groups and treatments were regarded as significant at p < 0.05.

### Data availability

All data generated or analysed during the current study are available from the corresponding author on reasonable request.

## Electronic supplementary material

Supplementary figures
