# Supplementary material for: Honokiol protects against doxorubicin cardiotoxicity via improving mitochondrial function in mouse hearts
Source: Sci Rep. 2017 Sep 20;7:11989. doi: 10.1038/s41598-017-12095-y (PMC5607346; doi:10.1038/s41598-017-12095-y)

Honokiol protects against doxorubicin cardiotoxicity via improving mitochondrial function in mouse hearts

Lizhen Huang[1,2], Kailiang Zhang[2], Yingying Guo[2], Fengyuan Huang[3], Kevin Yang[3], Long Chen[4], Kai Huang[4], Fengxue Zhang[1], Qinqiang Long[2, #], Qinglin Yang[2, 3, #]

[1] School of Basic Medicine, Research Center of Integrative Medicine, Guangzhou University of Chinese Medicine, Guangzhou, China 510006

[2] Division of Cardiology, Department of Internal Medicine, Tongji Hospital, Tongji Medical College, Huazhong University of Science and Technology, 1095 Jiefang Ave, Wuhan, China 430030

[3] Department of Nutrition Sciences, University of Alabama at Birmingham, AL, USA 35205

[4] Department of Cardiovascular Diseases, Union Hospital, Tongji Medical College, Huazhong University of Science and Technology, Wuhan, China, 430022

[#] Correspondence to:

Qinglin Yang, MD, Ph.D., Department of Nutrition Sciences, University of Alabama at Birmingham, 1675 Univ. Blvd., Webb429, Birmingham, AL, USA 35205, qyang@uab.edu

Qinqiang Long, PhD, Division of Cardiology, Department of Internal Medicine, Tongji Hospital, Tongji Medical College, Huazhong University of Science and Technology, 1095 Jiefang Ave, Wuhan, China 430030, qqlong@tjh.tjmu.edu.cn

Huang L *et al*. Figure S1

Full-length images of western blots presented in Fig. 3D, F. The cropped images shown in Fig. 3D, F are marked here with a red dotted line.

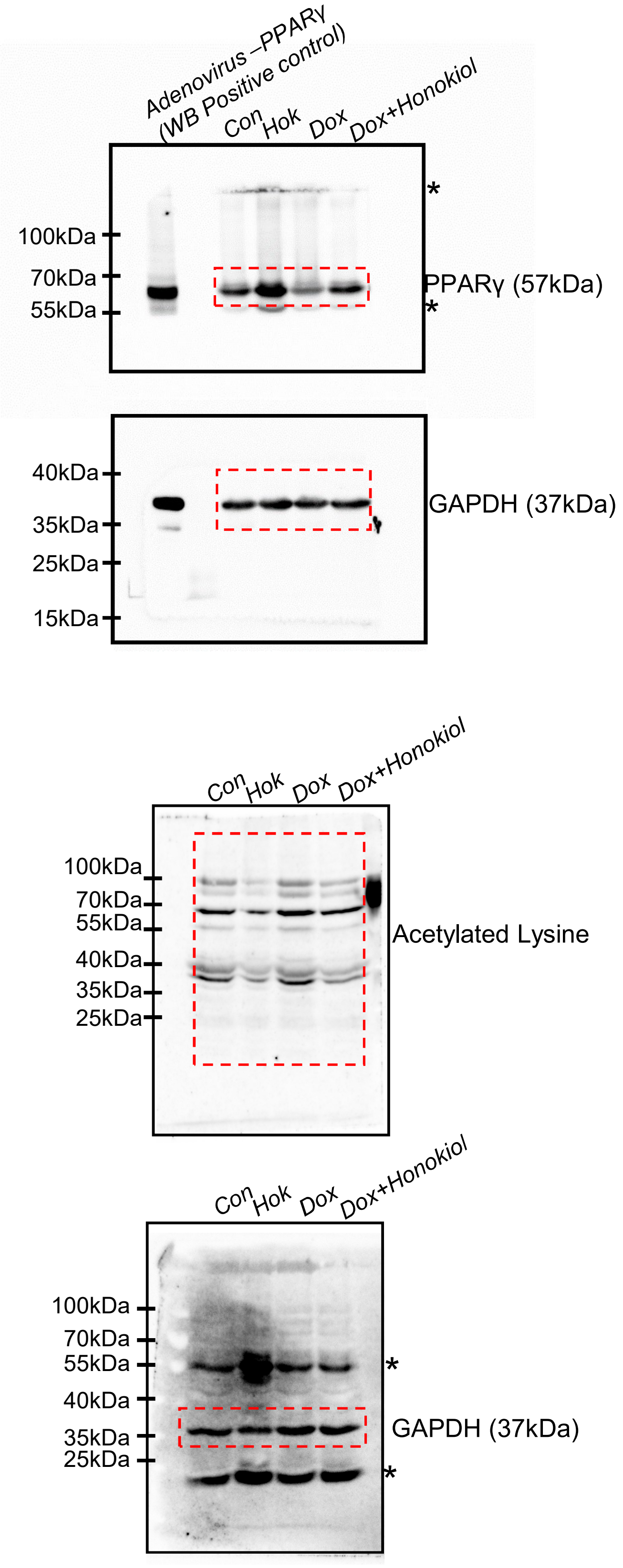

Huang L *et al*. Figure S2

Full-length images of western blots presented in Fig.8C. The cropped images shown in Fig.8C are marked here with a red dotted line.

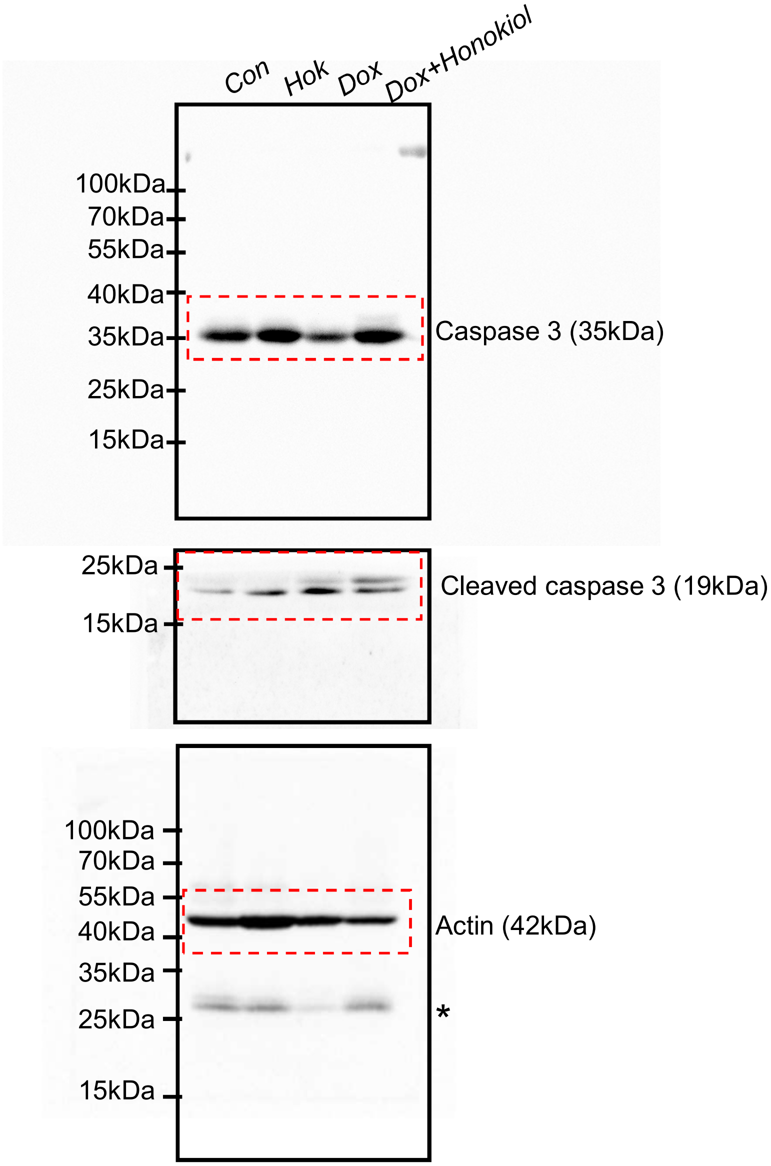

Supplement: Supplementary file 1 — Supplementary figures [file 41598_2017_12095_MOESM1_ESM.pdf]
